# Structuring for Culture: Exploring Organizational Preconditions for Culturally Appropriate Dementia Care in Sweden-a Thematic Analysis

**DOI:** 10.1177/23779608261494296

**Published:** 2026-09-28

**Authors:** Lisa Spang, Margaretha Norell Pejner, Annica Kihlgren

**Affiliations:** 1 School of Health Sciences, 6233 Örebro University, Örebro, Sweden

**Keywords:** Aging, Dementia, Nursing home, Migrants, Qualitative

## Abstract

Migration and demographic change have increased the number of people with dementia who live in secondary countries, intensifying the need for culturally attuned and person-centred dementia care. This study aimed to explore how staff and unit managers understand and enact cultural responsiveness in everyday care practices. 24 in-depth interviews were conducted with staff working in dementia care settings, and the data were analysed using reflexive thematic analysis. The results show a persistent organizational disconnect that limits the integration of cultural considerations into person-centred care. Participants described a common assumption that cultural identity becomes less relevant as dementia progresses, which contributed to cultural needs being overlooked in care planning and daily interactions. Staff reported lacking the knowledge, training, and structural support needed to work in culturally informed ways, despite expressing a desire to provide individualized care. Two overarching themes and four subthemes illustrate how organizational priorities, resource constraints, and implicit norms shape care practices. Staff highlighted challenges related to language barriers, unfamiliar traditions, and differing expectations around family involvement. They also described tensions between cultural adaptation and budget constraints, noting that efforts to personalize care were often overshadowed by the need to maintain “budget in balance.” Although some staff used their own cultural or linguistic knowledge to support residents, these efforts were informal and sometimes led to feelings of being overused as cultural intermediaries. Managers, in contrast, often viewed cultural matching based on ethnicity as a practical and cost-effective strategy, revealing differences in how cultural competence was understood across organizational levels. The findings demonstrate the need for stronger organizational structures to support culturally responsive dementia care, including targeted education, reflective spaces, and clearer guidelines. Strengthening institutional support may enable staff to better address cultural needs and uphold person-centred values in increasingly multicultural care environments.

## Introduction

As societies become increasingly intercultural, healthcare systems are challenged to ensure that care delivery is culturally appropriate, person-centered, and responsive to diverse needs (Shatnawi et al., 2023). In dementia care, this challenge is particularly pressing due to the vulnerability of the population and the complexity of care needs (Mesas-Fernández et al., 2024). However, barriers to cultural inclusiveness persist, including language difficulties, lack of culturally sensitive healthcare, and limited culturally appropriate care supports. Recent research substantiates these claims (Gaviola et al., 2024), highlighting a general lack of methodological rigor in studies examining dementia care for individuals from culturally and linguistically diverse backgrounds in nursing home settings. These findings point to a significant gap in the evidence base and underscore the need for further well-structured research to inform inclusive care strategies.

## Review of Literature

“Leininger’s Theory of Culture Care Diversity and Universality” (Leininger & McFarland, 2010) offers a foundational framework for understanding how cultural values, beliefs, and practices influence health and care experiences. The theory emphasizes the importance of recognizing both cultural diversity and commonalities in care needs and it advocates for the integration of cultural knowledge into nursing practice to promote culturally congruent care. Emerging scholarship highlights the current limitations of initiatives to promote cultural congruent care, such as limitations of traditional, checklist-driven models of cultural competence in dementia care for older migrants. Such approaches risk oversimplifying cultural identities and reinforcing stereotypes (Hiam et al., 2019). In response, several studies suggest more reflexive and critical frameworks grounded in cultural humility, which emphasizes ongoing self-reflection and awareness of power imbalances in care relationships (Russell, 2021). Scholars also draw attention to the role of marginalizing migrant older adults within care systems, underscoring the need to recognize and counteract exclusionary practices (Hiam et al., 2019). Furthermore, anti-racist practice is highlighted as essential for actively challenging structural inequities embedded in dementia care (Mohamed & Modanlo, 2025). To provide culturally appropriate care, cultural competence is required. This is achieved by the staff through a process consisting of acquiring cultural knowledge, cultural skills, cultural awareness, cultural encounters and cultural desire. This does not mean creating stereotypes from different countries but rather it means meeting the individual in their cultural context. (Campinha- Bacote, 2011). Therefore, in this study we use a culture competence perspective.

Despite its relevance, this perspective has not yet been firmly established within the Swedish healthcare system. This lack of cultural competence in Swedish dementia care may be partly explained by findings from a registry-based study using data from the Swedish Dementia Register (Lindgren et al., 2021). The study revealed that foreign-born individuals were significantly younger at the time of diagnosis, had lower MMSE scores, and were less likely to receive a specific dementia diagnosis compared to Swedish-born individuals. These differences were more pronounced among those with lower socio-economic status, who also received less home-based care. Notably, although the five largest migrant groups in Sweden are from Syria, Iraq, Finland, Poland, and Iran (Statistics Sweden, 2025), 84% of the foreign-born individuals with dementia were born in Nordic or other European countries regions with minor cultural differences from Sweden (Lindgren et al., 2021). This demographic composition may have contributed to the limited impetus for transitioning towards care with cultural competence.

The Swedish National Board of Health and Welfare estimates that there are 130,000–150,000 individuals with dementia in Sweden; the yearly incidence is 20,000–25,000 (The National Board of Health and Welfare, 2022). In 2020, there were 2.0 million residents registered with another country of birth in Sweden, accounting for 20% of the population (Statistics Sweden, 2021). In 2070, it is expected that nearly one third of the elderly residents in Sweden are born in another country than Sweden. The majority of foreign-born people in Sweden are from countries outside of Europe (The National Board of Health and Welfare, 2018;The National Board of Health and Welfare, 2022). The National Board for Health and Welfare highlighted in a report in 2018 that care of foreign-born people as a specific issue which needed to be further addressed, When the Government Offices of Sweden presented their dementia strategy for the years 2025-2028, efficient care for foreign-born persons with dementia was still stated as an issue which needed to be further investigated (Government Offices of Sweden, 2025).

As the aging population becomes increasingly diverse, there is a pressing need to culturally adapt dementia care to ensure it is equitable, and responsive to the unique values, beliefs, and needs of individuals from varied cultural backgrounds. Therefore, it is essential to investigate how cultural competence is currently integrated into the care of older adults with dementia living in nursing homes. By acquiring up-to-date knowledge on cultural encounters in care practices, it becomes possible to identify and reassess how cultural care is delivered to meet the diverse backgrounds of residents. This, in turn, can support the co-creation, structuring, and delivery of care. Consequently, the aim of this study was to explore how existing organizational structures and conditions within contemporary Swedish dementia nursing homes meet the needs of older persons from different cultural backgrounds.

## Methods

A qualitative design was used in the study and followed the guidelines according to COREQ (Tong et al., 2007). Data was collected through semi-structured interviews conducted between December of 2024 and May 2025. Inclusion criteria were to be either a unit manager or staff from one of the ten nursing homes specializing in dementia care which was recruited to a larger project called “Rethinking Environments in Dementia Care Homes (REDem)- paving the way for equality in care delivery”. The interviews were subsequently analyzed using the thematic analysis approach developed by Braun and Clarke (2022).

### Study settings and background of the participants

In Sweden, the municipality has responsibility up to and including nursing level for the care of people with dementia. There is also the possibility for private actors to operate dementia housing with agreement with the municipality (Health and Medical Services Act SFS 2017:30). Individuals with dementia may receive nursing home placements, depending on their needs and disease progression (Ministry of Social Affairs, 2001). Nursing homes are typically small-scale, homelike units designed to provide person-centered care in a safe and supportive environment (Sanford et al., 2015). Care is primarily delivered by care aides (CA) or nurse assistants (NA), with support from Registered nurses (RN), physiotherapists, and occupational therapists (OT) as needed.

This study was a part of the REDem research project. Within that project, ten Swedish nursing homes specialised in dementia care participated. As a part of a purposive sample, unit managers and staff at the nursing homes were invited to participate. A total of 24 participants, 11-unit managers and 13 staff were included in the study from ten different dementia nursing homes across four Swedish municipalities. Of the nursing homes, nine were run by the municipality and one by a private concern. The number of beds in the nursing home varied with a range from 24-174 (mean 65, median 52). Both unit managers and staff from each nursing home were recruited. Participants reported a wide range of experience in dementia care, with years of experience ranging from 3 to 30 years (mean: 13.7 years). Their tenure at their current nursing home ranged from 2 to 17 years, with an average of 6.3 years.

### Data collection

All participants including unit managers and staff were invited to participate in deliberative semi-structured individual interviews conducted in a separate room at their workplace during working hours, through a digital video call (Teams). The interviews were guided by an interview protocol comprising four themes: physical environment, social environment, communication, and organization (See supplementary; Interview Guidelines). The interviews were performed by author (AK) and (MNP), who are both experienced qualitative researchers with experience of interviewing. During the interviews, participants were encouraged to elaborate on their reflections related to these themes. They were also asked follow-up questions to explore how they addressed these themes in their daily interactions with individuals from culturally and linguistically diverse backgrounds. The interviews were recorded and lasted between 20-33 minutes and were collected between December 2024 and May 2025.

### Ethics approval and consent to participate

Ethical approval was obtained from the Swedish Ethical Review Authority (Dnr 2024-01131-01). All participants were given verbal and written information about the aim of the study and their right to, without any need for explanation, withdraw from the study at any time. All participants signed a consent form before participating in the data collection.

### Data analysis

The analysis was conducted following the process of reflexive thematic analysis as described by Braun and Clarke (2022). All authors, three female qualitative researchers (ranging in age from 37-67 years, with different experiences in dementia care as CA and RN and having Swedish and Spanish cultural backgrounds) who are experienced in qualitative research, contributed to the analytical process through triangulation (Patton, 2015). This approach was applied from the outset, as the second and last authors conducted the interviews and had a strong contextual understanding of the data. The first author transcribed the interviews and conducted a preliminary analysis, progressing from familiarization with the material to initial coding and the development of potential themes. The entire analytical process, including the preliminary themes, was then presented to the research team for discussion, validation and thereby triangulated. This meant that all authors had access to all interviews, both videos and transcriptions. During the analysis process five analysis meetings were held. Prior to these meetings, the authors prepared themselves by using the original data, and discussions were held on how the data corresponded with the initial codes, and on theme development. During these discussions, questions were asked such as ‘What do these similar experiences mean?’, ‘What are the staff describing that the unit managers are not?’, ‘How do the descriptions complement each another?’, and ‘What stands out?’ Following consensus, the final themes were named, and a draft of the findings was produced. An overview of the analysis process and resulting themes is presented in Table 1, Illustration of the analysis process, code to main theme.

**Table 1.** Illustration of the analyzing process, code to main theme

Examples of codes	Tentative analysis of content in themes	Subtheme	Theme	Main theme
➢ Celebrate different holidays not only Swedish	➢ Finding ways to create cultural familiarity in everyday life	*Reflecting on current adaptations to meet daily challenges in culture care*	***Informal Strategies for Cultural Adaptation in Dementia Care***	**Organizational Disconnect in Culturally Appropriate Dementia Care**
➢ Include family members to understand the older adult better				
➢ Offer food from different cultures				
➢ Music/Film/TV series from different countries				
➢ Have theme weeks from different cultures				
➢ Methods to translate languages, facilitate communication				
➢ Organise residents more by age and ethnicity to create more community.	➢ Organizational opportunities to further develop culturally appropriate dementia care	*Suggested areas for improvement to achieve culturally appropriate care*		
➢ Involving staff in management changes				
➢ Create education materials about cultural care.				
➢ Develop co-operation between different actors to adapt to culture.				
➢ Develop team meetings to discuss culture.				
➢ Innovative ideas that challenge old ways of working				
➢ Focus is not on culture but on disease or the need for care.	➢ Reveals the lack of cultural competences.	*The Perceived Decline of Cultural Relevance in Advanced Dementia*	***The Impact of Cultural Incompetence on Care Perceptions***	
➢ Language is important, but it does not have to be the basis of a good relationship	➢ Diminishing view of the importance of culture			
➢ Advantages and disadvantages of having a multicultural staff	➢ Attempts to use language skills within the organization.	*The Limits of Cultural Responsiveness in Dementia Care*		
➢ Best fit within budget	➢ Best care on a budget			

## Results

The findings were unified by one main thematic thread “*Organizational disconnect in culturally appropriate dementia care*” with two underpinning themes and four subthemes. This thematic thread is illustrated in Table 2, Final thematic map.

**Table 2.** Final thematic map

Main Theme	Organizational disconnect in culturally appropriate dementia care			
*Theme*	*Informal strategies for cultural adaptation in dementia care*		*The impact of cultural incompetence on care perceptions*	
Subtheme	Reflecting on current adaptations to meet daily challenges in culture care	Suggested areas for improvement to achieve culturally appropriate care	The perceived decline of cultural relevance in advanced dementia	The limits of cultural responsiveness in dementia care

### Organizational disconnect in culturally appropriate dementia care

The main theme, “Organizational disconnect in culturally appropriate dementia care,” captures the gap between formal organizational structures and the practical realities of delivering culturally attuned care. The organizations were judged to contain inefficient working methods and a lack of operational routines, which led staff to create their own. This disconnect is explored through two interrelated themes. The first, “Informal strategies for cultural adaptation in dementia care,” highlights how frontline staff often take the initiative to create space for cultural responsiveness in the absence of formal guidance or institutional support. Within this theme, the subtheme “Reflecting on current adaptations to meet daily challenges in culture care” illustrates how staff navigate and adjust their practices to address emerging cultural needs, while “Suggested areas for improvement to achieve culturally appropriate care” points to the need for more structured and systemic approaches. The second theme, “The impact of cultural incompetence on care perceptions,” addresses how a lack of cultural competence influences both staff and managerial perceptions of care quality. This is further addressed through the subtheme “The perceived decline of cultural relevance in advanced dementia,” which reflects the belief that cultural identity becomes less significant as the disease progresses, and “The limits of cultural responsiveness in dementia care,” which underscores the complexity of adapting care to meet the diverse cultural backgrounds of all residents. Together, these themes reveal the tensions and adaptations that shape culturally responsive dementia care in practice.

### Informal strategies for cultural adaptation in dementia care

This theme sheds light on the creative and often improvised efforts made by care staff to address cultural needs in the absence of formal organizational support. Rather than relying on structured guidelines, staff members draw on personal experience, intuition, and collaboration to navigate cultural complexities in everyday care. These informal strategies reflect a deep commitment to person-centered care yet also expose the vulnerability of a system that places the burden of cultural adaptation on an individual level, e.g., staff rather than on the organization.

### Reflecting on current adaptations to meet daily challenges in culture care

Staff described using a range of informal strategies in addressing the cultural needs of residents in everyday care, in relation to traditions, family involvement, provision of familiar food, playing different music and using translation tools. One common strategy involved keeping traditions, the celebration of culturally diverse holidays, moving beyond traditional Swedish customs such as Midsummer to include festivities from other cultures. This was seen to acknowledge and validate residents’ cultural identities.

*“It’s clear that everyone enjoys celebrating, and relatives often help us organize everything according to the person’s traditions*.” *Quote from Staff*

Family members were highlighted as essential partners in care, often contributing valuable insights into residents’ life stories, preferences (e.g., only allow support with personal hygiene from male or female staff), and routines. Their involvement extended beyond emotional support, with some families regularly translating foreign languages, bringing culturally familiar food or even staying overnight in residents’ apartments due to the lack of dedicated guest facilities. Staff expressed a desire for more formal spaces to accommodate visiting relatives, recognizing their role as part of the care team. In addition to letting family members translate, the staff also used translation tools, such as Google Translate, and the creation of simple phrase lists were also mentioned as practical ways to help bridge language gaps.

*“For example, we may not understand what a resident who speaks Polish is saying, and the translation app on the phone doesn’t understand her either. So, we call a family member who speaks her language, but the resident often just repeats the same incoherent phrases, and her relatives can’t understand what she wants either.” Quote from Staff*

Food and meals emerged as a central aspect of cultural adaptation. While organizational constraints limited the ability to provide personalized meals, staff made efforts to accommodate dietary preferences where possible. In cases where residents rejected standard meals, families often stepped in to provide culturally appropriate alternatives. The importance of taste, familiarity, and cultural meaning in food was repeatedly emphasized.

“*You know, we order our food from a kitchen that only provides Swedish home cooking, but the staff are amazing. They bake and cook dishes from the person’s own culture.” Quotes from unit manager*

Other adaptations included the use of music, television, and film in residents’ native languages, accessed through personal streaming services or staff members’ own devices. These tools were used to create a sense of familiarity and comfort, especially for residents with limited Swedish language skills. Together, these reflections illustrate a strong commitment among staff to adapt care practices in culturally responsive ways, despite limited organizational support. The adaptations were often improvised and dependent on individual initiative, highlighting both the creativity and the structural limitations within organizational support from the current care system.

### Suggested areas for improvement to achieve culturally appropriate care

Staff identified several key areas where dementia care could be improved to better reflect and respond to cultural diversity. A prominent suggestion was to organize residents more thoughtfully by age and ethnicity, with the aim of fostering a stronger sense of community and shared experience. Staff noted that when residents share cultural or generational backgrounds, it can enhance social interaction and reduce feelings of isolation.

“*Today, individuals are offered a placement in a nursing home based on diagnosis and this results in a mix of very different people, but what does a 60-year-old have in common with a 90-year-old? Units should be organized according to demographic characteristics, including age as well as cultural and linguistic background.” Quote from Staff*

They also requested educational materials and structured training on cultural care, to develop the staff’s skills in how to interact with residents from different cultures. Staff expressed a desire for clearer guidelines for culturally responsive practices, and a shift away from rigid routines that may overlook individual and cultural differences.

*“Of course, it would be better to have clear guidelines from the organization, but … that’s not how things are at the moment” Quote from Staff*

By taking up more in-depth practical learning opportunities that go beyond surface-level workshops, they desire to build on their everyday approach. The need for education was congruent with the gap of knowledge within cultural care, identified by unit managers. However, the unit managers had no idea how to create or find such educational material.

*“I don’t have that training or expertise, and the question is who should provide it” Quote from unit manager*

Unit managers also highlighted the need for stronger collaboration between different actors, including care teams, management, families, and external organizations such as cultural organizations and associations. Such cooperation was seen as vital for adapting care environments and routines to better reflect residents’ cultural needs, particularly in cases where language barriers or unfamiliar customs create distress. In addition, there was a clear appetite for innovative ideas that challenge traditional ways of working. Staff encouraged a more flexible and open-minded approach to care, where innovation is welcomed and assessed. This included rethinking routines, environments, and even dietary offerings to better align with residents’ cultural preferences.

### The Impact of Cultural Incompetence on Care Perceptions

This theme explores how limited cultural competence within dementia care settings shapes both the unit managers and staff’s perception and delivery of care. A recurring pattern among unit managers was the tendency to prioritize medical needs and basic care routines over cultural considerations, reflecting a broader view that culture becomes less relevant as dementia progresses. While language was acknowledged as important, it was not always seen as essential for building meaningful relationships, suggesting a diminished emphasis on cultural identity in care interactions. Together, these findings reveal a care environment where cultural competence is inconsistently valued and insufficiently supported, ultimately affecting how care is perceived and experienced by both staff and residents.

### The Perceived Decline of Cultural Relevance in Advanced Dementia

Unit managers described how they did not talk about culture, as they always collaborated with a person-centered approach. Nevertheless, staff described how, in later stages of dementia, the focus tends to shift away from cultural identity and towards ensuring basic care needs are met, such as nutrition, hygiene, and safety.

“*When people reach a certain stage of dementia, culture doesn’t matter anymore. They´re all very much the same.” Quote from Staff*

Language loss was frequently mentioned as a complicating factor. Many residents revert to their native language or lose verbal communication altogether, making it difficult to maintain culturally specific interactions. However, staff emphasized that language is not always the foundation of a meaningful relationship. Instead, trust, continuity, and non-verbal communication, such as body language, tone, and routine were seen as more critical in building connection and understanding.

*“It’s about having the time to sit with them, putting a hand on their shoulder, and using simple words or phrases in their language”. Quote from Staff*

Nevertheless, staff demonstrated a strong commitment to person-centered care, often using creative methods such as life stories, photographs, and family input to understand and respond to residents’ backgrounds. These efforts, however, were informal and dependent on individual initiative rather than organizational support. By using these person-centered approaches, they defended them self on not adapting to culture, as they could not understand how it could contribute to the residents’ state of mind.

### The Limits of Cultural Responsiveness in Dementia Care

While some staff expressed a strong willingness to adapt care to residents’ cultural backgrounds, they also acknowledged the complexity of meeting diverse needs within the realities of shared environments, limited resources, and standardized routines. In settings where residents represent a wide range of nationalities, staff noted that it becomes increasingly difficult to tailor care in a way that feels meaningful to everyone. One key challenge was the balance between cultural adaptation and budget constraints. Staff frequently referred to the pressure of maintaining “budget in balance,” which often overshadowed efforts to personalize care.

“*My job as a unit manager is to provide best possible care…. But within the budget of course.” Quote from unit manager*

Although there was openness to using language skills and cultural knowledge within the team, these efforts were often informal and lacked structural support. Some staff even reported feeling overused when asked to act as cultural or linguistic intermediaries, especially in cases when cultural differences within the same language group created tension.

*“People think that if you speak the same language, you automatically understand each other’s culture. But even within the same language group, there can be huge differences, and that can create disagreements. Quote from staff*

Staff emphasized that cultural competence cannot be assumed based on ethnicity alone, which unit managers on the other hand described as successful and cost-effective approach to meeting the cultural needs of residents.

## Discussion

By exploring the existing organizational structures and conditions within contemporary Swedish dementia nursing homes, an organizational disconnect in culturally appropriate dementia care was identified as the main theme in this study. Even though unit managers claim to provide flexible and person-centered care for all their residents including individuals with cultural and linguistically diverse backgrounds, their staff contradict this by describing how it is because of their initiative and person-centeredness that cultural adaptations take place in their nursing home unit. Within this context, staff approached culture in different ways, although these approaches were shaped by their individual levels of knowledge. This shows how cultural competency (cultural knowledge, cultural skills, cultural awareness, cultural encounters and cultural desire) occur at an individual level and does not come from the organization.

Previous research increasingly supports person-centered care as not only an ethical approach but also an evidence-based strategy that significantly enhances the well-being of individuals living with dementia. By focusing on the individual’s needs, preferences, and life history, person-centered care interventions have been shown to reduce agitation, neuropsychiatric symptoms, and depression, while improving overall quality of life (Fazio et al., 2018; Kim & Park, 2017). Although person-centered care is an established approach in care, the discussion around person-centered care is not merely compassionate care, it is about designing effective, long-term solutions that meet the complex needs of individuals from cultural and linguistically diverse backgrounds with dementia. Even though the unit managers and staff claim to provide person-centered care, the findings in our study suggest a lack of cultural competence in how care is structured and delivered. Cultural desires were often overlooked or seen as secondary, particularly when they were not explicitly expressed. It also suggests a need to reframe cultural care not as optional or secondary, but as an integral part of person-centered dementia care, even in its most advanced stages. According to Leininger’s Theory of Culture Care Diversity and Universality (Leininger & McFarland, 2010), truly effective care must be culturally congruent aligned with the patient’s cultural values, beliefs, and practices. One of the key limitations of person-centered care is that it tends to focus on the individual in isolation, without fully considering the cultural systems that shape their identity and care expectations. Leininger argues that care which ignores cultural background risks being superficial or even inappropriate, particularly in multicultural settings. To achieve more culturally appropriate care in dementia homes, unit managers need to recognize that although they say they deliver care with a person-centered care focus, staff highlight how they lack informal organizational support in the day-to-day delivery of culturally appropriate care. The constraints of only offering Swedish home cooking, not distributing access to streaming media services and not providing them with appropriate translation tools, leaves them to solve cultural adaptation based on their own initiatives within the staff groups. Further, these organizational disconnects are reinforced by economic conditions and an often-strained budgets. The necessity of balancing budgetary constraints and cost-effective operational strategies with the principles of person-centered care has previously been identified as a key organizational challenge for first-line managers within home-based care in Sweden (James et al., 2024). Our findings confirm this observation and further suggest that this tension is also evident in nursing home settings. This challenge can be understood because of top-down organizational structures, which, as previous research has demonstrated, often function as barriers within healthcare systems. These systems are typically characterized by a task-oriented approach, limited time allocation, and strict financial oversight (Hagerman et al., 2019; Strömberg et al., 2019). Moreover, studies have shown that organizational resources are frequently insufficient to meet the complex demands of elderly care (Hagerman et al., 2019; Kihlgren et al., 2021; Silverglow et al., 2021). As a result, the prioritization of cost-effectiveness tends to overshadow fundamental ethical principles such as human dignity and solidarity with those in need.

Another gap found in the results of this study is the lack of structured education in cultural competence for care staff (Campinha- Bacote, 2011). Drawing on the work of recent research Hiam et al. (2019); Mohamed & Modanlo (2025); Russell (2021), argue that cultural competence education must move beyond checklist-based cultural competence which risks stereotyping and oversimplifying older migrants’ identities. Rather than providing prescriptive lists of “cultural facts,” organizations could strengthen support by fostering ongoing dialogue, reflective practice, and collaborative problem-solving around cultural dilemmas that arise in everyday care. This includes offering supervision formats where staff can discuss uncertainties, share experiences, and explore how cultural identity intersects with dementia progression, communication challenges, and family involvement. Such approaches encourage cultural humility and critical reflection without reducing culture to a set of predefined categories. In this way, organizational support can enhance staff confidence and competence while avoiding the oversimplification and stereotyping that checklist models risk reinforcing. Similar approaches are needed in Swedish dementia care to strengthen staff training and improve culturally responsive care for an increasingly diverse older population. While person-centered care focuses on individual preferences and life histories, culturally competent care requires staff to recognize how cultural identity shapes those preferences and to adapt care practices accordingly. Developing this distinction further would help organizations ensure that person-centered care does not become culturally neutral, but instead actively incorporates cultural meaning, values, and social context into everyday decision-making.

While person-centered care encourages empathy and responsiveness, it does not always equip professionals with the tools to assess and respond to cultural diversity in a systematic way (Kim & Park, 2017). Leininger’s model, which recognizes both commonalities and diversity, emphasizes the need for cultural assessment frameworks and training to ensure care is both person- and culture-centred (Leininger & McFarland, 2010). Therefore, a conclusion might be for the management of dementia care on a municipal and county level. To develop educational materials on cultural competence among their residents and how emphasizing it is important to understand someone’s culture, to be fully person-centered.

### Strengths and Limitations

A key strength of this study lies in its use of semi-structured interviews, which allowed for in-depth exploration of participants’ experiences and perspectives within dementia care. The inclusion of both staff and unit managers from diverse educational and professional backgrounds across multiple municipalities enhanced the richness and transferability of the data. Furthermore, the study was enhanced using thematic analysis performed by triangulation to strengthen the credibility and rigor of the findings. The researchers had no clinical positions and no previous relationships with the participants. The transferability is strengthened as the data was collected in four different regions of Sweden and represents both bigger cities and smaller towns, both nursing homes run by private actors and by the municipalities.

However, some limitations should be acknowledged. Conducting interviews via digital video calls may have affected the depth of interaction and limited the ability to capture non-verbal cues. The data collection could have been strengthened with observations of the everyday care performed at the nursing home units, to validate the experience expressed by staff and unit managers. Additionally, the length of the interviews may be considered too short to be regarded as in-depth. However, the aim was not to capture in-depth descriptions but to explore experiences. The interviews in total contained 467 minutes of responses relevant to the objective of the study, which resulted in the author’s judging them to be sufficient in terms of achieving information power (Braun & Clarke, 2021).

## Implications for practice

These findings suggest that dementia care organizations must move from general aspirations of cultural responsiveness to concrete operational changes which strengthen staff competence and reshape everyday decision-making. A key implication is the need for systematic assessment of cultural competence for nurses and frontline care staff, to enable unit managers to identify specific gaps in knowledge, communication skills, or confidence and to provide targeted training rather than relying on individuals’ initiatives. Cultural competence should be treated as an enforceable organizational requirement, supported through structured education, supervision, and access to culturally relevant resources. The findings also illustrate how cultural considerations can influence practical decisions, such as resident placement. Instead of assigning residents solely based on care level or available beds, organizations should incorporate factors such as age, gender, ethnicity, and cultural background to create living environments that feel familiar, safe, and socially coherent. Embedding these practices into routine care planning, team discussions, and managerial oversight can help ensure that cultural identity becomes a consistent and meaningful component of person-centered dementia care.

Finally, nurses with cultural or linguistic knowledge should be protected from informal role overload; formal access to interpreters or designated cultural mediators can reduce the risk of being overused as cultural or linguistic intermediaries.

## Conclusions

To close the gap between formal organizational structures and the practical realities of delivering culturally attuned care, management must provide practical support for culturally responsive practices. This includes offering culturally and linguistically diverse focused educational materials and resources, which would enable staff to deliver truly person-centered care that respects residents’ cultural identities. Staff should not be left to rely solely on their personal initiative to meet cultural needs. Instead, care organizations must embed cultural competence into daily routines and decision-making. By being prepared to rethink and adapt strategic intentions, dementia care can become more inclusive, consistent, and effective.

## Supplemental Material

Supplemental Material - Structuring for Culture: Exploring Organizational Preconditions for Culturally Appropriate Dementia Care in Sweden-a Thematic Analysis Supplemental material for Structuring for Culture: Exploring Organizational Preconditions for Culturally Appropriate Dementia Care in Sweden-a Thematic Analysis by Lisa Spang, Margaretha Norell Pejner and Annica Kihlgren in Sage Open Nursing.

## Data Availability

The datasets on which the conclusions in this article are based are not publicly available due to ethical and legal restrictions. Consequently, access to the data is available only upon request, following ethical approval from the Swedish Ethical Review Authority, that restricted access to the data for Dnr 2024-01131-01.*
