# Supplementary material for: Structuring for Culture: Exploring Organizational Preconditions for Culturally Appropriate Dementia Care in Sweden-a Thematic Analysis
Source: SAGE Open Nurs. 2026 Sep 28;12:23779608261494296. doi: 10.1177/23779608261494296 (PMC13769926; doi:10.1177/23779608261494296)
Supplement: Supplementary file 1 [file sj-pdf-1-son-10.1177_23779608261494296.pdf]

INTERVIEW GUIDELINES

Four themes, physical environment, social environment, communication, and organization was covered semi-structed by six main questions followed by individual follow-up questions to facilitate conversations to explore how existing organizational structures and conditions within contemporary Swedish dementia nursing homes meet the needs of older persons from different cultural backgrounds.

1. Can dementia specialized care unit be adapted to the individual's needs from a multicultural perspective?
   - How do you communicate with residents with different languages?
   - What do you know about the residents' cultural preferences?
   - How do you cater to their needs?

2. How could indoor communal areas be adapted from a multicultural perspective?
   - Can you give an example of one time you have adapted communal areas in the past?

3. Do you hold formal team meetings where you discuss the individual's needs?
   - How do you communicate the residents' needs with all of the staff?

4. Do staff have the flexibility to adjust the day's schedule to meet the individual's needs for that day or at that moment?
   - Can you tell me have a normal day could look like?

5. How could outdoor communal areas be adapted from a multicultural perspective?
   - What do cultural adaption mean to you according to adapted outdoor communal areas?

6. What flexibility does the organization allow the department to meet the individual's needs?
   - Do you have the means that you need to meet the individual needs?
